# The role of hand fingerprints on predisposition of cancer development

**DOI:** 10.1016/j.heliyon.2023.e14074

**Published:** 2023-02-26

**Authors:** Sakineh Abbasi, Seyed Mohammad Ayyoubzadeh

**Affiliations:** a Department of Laboratory Science, School of Allied Medical Sciences, Tehran University of Medical Sciences, Tehran, Iran; b Department of Health Information Management, School of Allied Medical Sciences, Tehran University of Medical Sciences, Tehran, Iran

**Keywords:** Dermatoglyphics, Fingerprints, Cancer

## Abstract

Fingerprints or dermatoglyphics contain patterns that were formed by parallel ridges on the bare skin of fingertips**.** This property on the skin, especially on the finger, makes it possible to hold objects with our fingers, and this feature can also be used to determine identity. After cardiovascular diseases, cancer is the second cause of death worldwide. In this paper, we reviewed the associations reported between fingerprint patterns (dermatoglyphics) and cancer types. In this review, we focused on six types of cancer, including gynecological cancers, oral cancer, prostate cancer, gastric cancer, leukemia, and pituitary tumors, and their connection with fingerprints. The dermatoglyphic could be a potentially useful tool for early diagnosis of predisposition in developing some diseases. As some patterns inform us about leading to deadly diseases, such as cancer, which could be prevented, or at least by early diagnosis and taking proper care, the mortality rate could decline. Thus, the fingerprints that have been primarily observed in particular cancers require more research.

## 1 Introduction

Dermatoglyphics (dermis = skin, glyphs = engraving) is a kind of dactylography based on the study of the layer of protrusions and their configuration in the human fingertip and palm or even toes and soles of the feet [1]. The fingerprint or dermatoglyphics contains patterns formed by parallel protrusions on the bare skin of the fingertips [2]. It is a helpful device for searching conditions with a suspected genetic basis. Also, dermatologic patterns on the fingertips often differ in syndromes and other systemic details compared to the general population [3].

The fingerprint is created between the fifth and sixth week of embryonic development, fully formed by the 21st week, and remains unchanged [4]. This feature on the skin, especially the finger, makes it possible to hold objects with the fingers. It is also used as individual identification [5] and a noninvasive medical diagnostic tool [1].

**Fig. 1:**
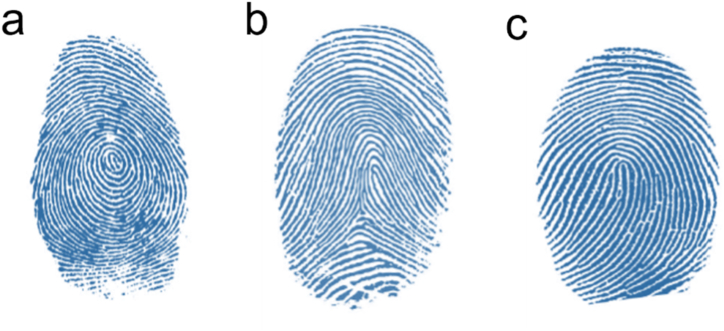
Three basic patterns. a) Whorl b) Arch c) Loop (Adapted with permission from Ref. [9]).

Francis Galton and Edward Henry [6] conducted the first research on fingerprinting and classification in 1892 and described that fingerprint classification includes three basic patterns Arch, Loop, and Whorl (Fig. 1). [7,8] The archaeologists have discovered fingerprints dating back to 1792–1750 BCE in pottery tablets in Babylon [2].

**Fig. 2:**
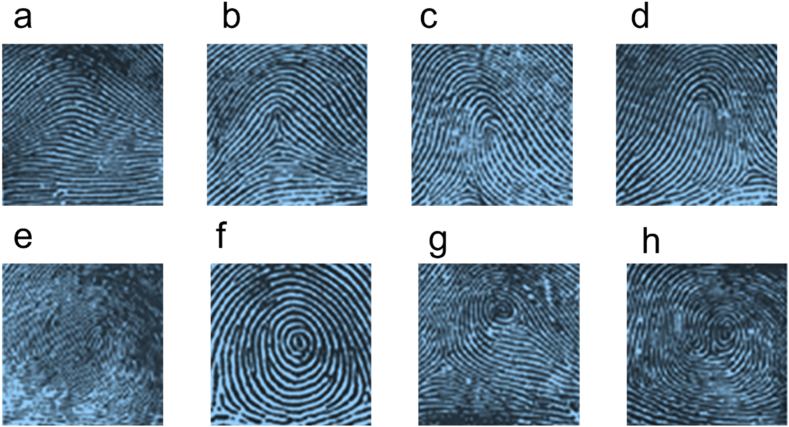
Eight basic patterns that the FBI uses. a) Plain arch. b) Tented arch. c) Ulnar loop. d) Radial loop. e) Double loop whorl. f) Plain whorl. g) Central pocket loop whorl. h) Accidental loop whorl (Adapted from Ref. [11]).

These patterns were later divided into eight main fingerprints that the FBI uses until now (Fig. 2). The arch pattern has two sub-types: Plain Arch and Tented Arch. Also, the loop pattern has two sub-categories: Radial Loops and Ulnar Loops, and for the pattern of the whorl, here are four sub-groups: Plain Whorl, Central Pocket Loop, Double Loop, and Accidental Whorl [10].

Cancers are one of the main causes of worldwide death [12]. Some studies suggest that dermatoglyphics analysis could be a tool for early diagnosing a patient's genetic tendency to a particular group of hereditary diseases [13,14].

Thus, in this study, we aimed to review the literature regarding qualitative characteristics of hand fingerprints' role in the predisposition of cancer development. We aimed to determine the patterns of fingerprints which has a relationship with cancer and identify the cancer types that could have an association with fingerprint patterns.

In the following, six types of cancer, including gynecological cancers, oral cancer, prostate cancer, gastric cancer, leukemia, and pituitary tumors, and their connection with fingerprints are described.

## 2 Gynecological cancers

We divided the studies on fingerprint patterns of gynecological cancers into two groups: 1. Breast cancer, 2. Cervical cancer. Generally, In 2018, Abbasi S. et al. Studied finger patterns in gynecological cancers in Iran. Dermatoglyphic analysis proved that patterns of loop and arch shifted significantly in patients compared to controls. Although the odds ratio shows that a loop pattern in 6 or more fingers might increase the risk factor in gynecological cancers [9].

### 2.1 Breast cancer

Breast cancer is the most prevalent cancer among women globally [15]. Abbasi S. et al. (2006) revealed that the Whorl pattern among Iranian breast cancer women was significantly more frequent than among the normal women population (48.7% vs. 27.5%) [16]. Recently, another study used this data to build more advanced models to predict breast cancer [17].

In 2009, in Nigeria, Oladipo G. S. et al. Reported that the ulnar loop showed a statistically significant association with mammary malignancy [18]. In 2010, Sridevi N. S. et al. Reported loops increased in both hands' finger patterns statistically in cases (66.5% in patients and 59.2% in normal individuals) among Indian women with breast cancer. However, Whorl and arch patterns decreased in patients versus normal individuals (30.3%, 34.0%, and 3.2%, 6.8%, respectively) [19].

In 2012, Lavanya J. and colleagues worked on groups of women with noninvasive markers of breast cancer in India. They found that whorl patterns were more in the cases group (39%) in comparison with the normal group (29%) [20]. In 2013, Rizada A. et al. Reported a significant increase in cancer patients in comparison to those in control for arch patterns (39.8% in patients vs. 16.2% in control) and decreasing in both radial loop and whorl patterns in cancer patients (36.8%, 20.2% vs. 50.4%, 30.8% respectively) [21].

In 2018 Mušanović J. and colleagues focused on quantitative analysis of fingertip patterns in a patient with breast cancer carcinoma in Bosnia and Herzegovina. They mentioned that arch and whorl patterns in breast cancer patients increased compared to normal individuals (28.7% vs. 25.2% and 22.8% vs. 14.4%, respectively); however, the loop patterns decreased in breast cancer patients versus normal individuals (62.4% vs. 78.5%) [22].

In 2019 Bin Thabit M. A. et al. Searched forensic dermatoglyphic traits and clinicopathological features in 68 Yemeni females with different breast cancer stages. In their research, the loops and whorls patterns were defined as having a significant association with breast cancer. Based on their study, the loop patterns (30.4%) in the little finger and whorl patterns (33.6%) in the index finger were higher than other patterns in the other fingers of patient cases [23]. In 2021, Dimitrova T. et al. Searched the role of dermatoglyphics in breast cancer. This research examines the relationship between fingerprints and cancer in different communities. For example, in India, patterns are increasing in cancer patients more than normal. The ulnar loop in the left hand represents 76.8% of the patient compared to 34.4% in normal individuals, and the ulnar loop represents 77% of patients compared to 34.6% in normal individuals. Besides, in both hands, whorls patterns showed significantly greater in breast cancer patients than in normal individuals (53.2% vs. 15.8% and 56.0% vs. 16.2%, respectively). The ulnar loop is smaller (34.4% vs. 76.8% and 34.6% vs. 77.0%, respectively) in 100 breast cancer Bulgarian women between 30 and 60 years old [24].

In 2021, in a systematic review, Inggarsih R. et al. from Indonesia reported that breast cancer patients have more whorl fingerprint patterns (42.80%) compared to (23.80%) controls. However, radial loop patterns (3% vs. 4%), ulnar loops (50.40% vs. 65.00%), and arches (3.80 vs. 6.40%) were decreased in comparison with the control group [25].

In summary, it can be concluded that the whorl pattern may be considered to be a factor in identifying the predisposition to breast cancer development in women in Iran, Bosnia and Herzegovina, and Indonesia.

### 2.2 Cervical cancer

Cervical cancer is a common disease and the fourth reason influencing mortality among women, with about 311,367 deaths reported in 2018 [26,27].

In 2016, in India, Sofia P et al. reported in their paper that for quantitative and qualitative analysis of dermatoglyphics, high repetition of rings and low frequency of ulnar rings in both hands and recurrence in the repetition of arches in the left hand were observed when comparing to the right (8.4% vs. 3.85) [28].

In 2018, Pramanik A. et al. Studied fingerprints in cervical cancer in 72 cases and compared them with 72 normal individuals in India. The radial loop in cases was less (3.19%) in comparison to the control (7.3%). Besides, the frequency of ulnar loop in cases (52.7%) was reduced in comparison to normal (60.3%) [29]. In 2022, Pravallika K. et al. Worked-on patterns in fingertips in women with cervix carcinoma in India. They found a strong relation between loop patterns and cervix cancer in 300 cases. Also, both hand whorl patterns showed a stronger positive correlation. They found 443 whorls and 1008 loops on the right hand and 425 whorls and 1043 loops on the left hand. At the same time, loop patterns are seen more than whorls in both hands. And there were 443 whorls and 1008 loops on the right hand, 425 whorls, and 1043 loops on the left hand [30].

In summary, it can be concluded that loop patterns may be considered to be a factor in identifying the predisposition to cervical cancer development in women in India.

## 3 Oral cancer

Oral squamous cell carcinoma (OSCC) is a cancer that involves the head and neck regions. Tobacco and alcohol are risk factors for oral cancer [31]. This cancer causes panic, holds an undeserved high ranking as a killer, and involves more than 90% of oral malignancies. In 2020 Vaishali S. et al. in India worked on 15 oral squamous cell carcinoma (OSCC) patients and dermatoglyphic patterns. Loop and arch patterns increased in OSCC patients versus control individuals (50%, 30% vs. 12%, and 20%, respectively). However, the whorl patterns decreased in OSSC patients (20% vs. 68%) [32]. In 2013, Gupta A. and his colleagues studied fingertips patterns in 90 Indian male patients (30 subjects had SCC) and 30 Indian male control. This study also revealed that the pattern of arches and loops increased in patients compared to control subjects (70%, 58.6% vs. 2.0%, 49.6%), whereas the whorl patterns significantly increased in control subjects (34.4% vs. 48.4%) [33]. Tonkaboni A. et al. Worked on 140 Iranian patients categorized into two 70-subjects. The first category has 36 OSCC males and 34 OSCC females, and the second category has 36 males and 34 females with any oral trauma as control. They found significant differences between OSCC and the control group for the arch pattern (6.7% vs. 5.0%). However, the differences between the OSCC group and control for loop and whorl patterns were insignificant (46.4%, 46.8% vs. 48.15, and 46.9%, respectively [34].

In September 2022, Venkatesh E. et al. in India studied fingertip patterns of hand association with OSCC in 30 patients compared to 30 controls. Again, they found that statistically, there were differences in arch and loop patterns in OCCS versus control individuals (7%, 60% vs. 2%, 30%, respectively), whereas the whorl patterns decreased (32% vs. 68%) [35]. Jetty D. et al., in May 2022, found the same results in the same ethnicity (Indian). For arch and loop patterns showed a significant increase in 30 OSCC patients versus 30 control individuals (60.7%, 33.3% vs. 28.6%, 30.6%, respectively). However, the whole pattern decreased (29.0% vs. 40.5%) [36].

In summary, it can be concluded that arch and whorl patterns may be considered to be a factor in identifying the predisposition to cervical cancer development in women in India.

## 4 Prostate cancer

Prostate cancer is the second most prevalent cancer among men and the fifth cause of death among cancer-related deaths globally [37].

In 2020 Mishra S. et al., in their research on 30 prostate cancer patients and 30 normal individuals from India, found that the percentage of the whorl, arch, and radial loop patterns in cancer patients was significantly higher compared to normal ones. In more detail, the ulnar loop and whorl patterns showed an increasing percentage in both hands in patients in comparison with control individuals (the whorl, arch, and radial loop patterns percentages in cancer patients were 37.17%, 17.11%, and 1.32%, vs. 30.67%, 13% and 1.07% in the normal individuals respectively) [8]. More interesting that in 2009, Oladipo G. S. and his colleagues found the same results and concluded that dermatoglyphic patterns in hands could be useful in the early diagnosis of prostate cancer [38].

In summary, it can be concluded that multiple patterns may be considered to be a factor in identifying the predisposition to prostate cancer development in men in India.

## 5 Gastric cancer

Gastric cancer is the third cause of cancer death and the fifth most prevalent cancer globally [39]. In 2017 Abbasi S. et al. Reported briefly that the whorl and loop patterns were the most patterns among Iranian gastric cancer patients. Whorl and loop patterns in more than six fingers were compared, 54.2% of cancer patients had more whorl patterns compared to control with 44.5%, and for loop patterns, 27.5% of patients had less than control with 46.2% [40].

## 6 Leukemia

Leukemias are malignant diseases of bone marrow and blood [41]. Bukelo M. J. in India 2011 studied dermatoglyphic patterns in 24 children, including Acute lymphoblastic leukemia (ALL) cases and 24 healthy children. The results showed whorl, and arch patterns increased in the index finger of the hand in comparison to control individuals (26%,7% vs. 15%, 4%)). However, loop patterns decreased in patient groups (15% vs. 29%) [42].

In 2017 Abd AL-Wahab S. and colleagues studied dermatoglyphics patterns in leukemia in Iraq. In this study, patients diagnosed with leukemia comprised 50 males and 50 females. The results showed that the number of arches and also ulnar loops increased in both hands in male leukemia patients (12.2% in patients vs. 6% in normal individuals and 42.2% in patients vs. 28.8% in normal individuals, respectively), whereas the whorl patterns (32.4% in patients, vs. 38.8% in normal individuals) and radial loops declined in both hands in male leukemia patients (13.2%in patient vs. 26.4% in normal individuals) in comparison with the healthy male. Although in a female with leukemia, the number of arch patterns and whorl patterns increased (14.4% in patients, 8% in normal individuals, 36.2% in patients, vs. 28.8% in normal individuals, respectively). The radial loop in female patients decreased (14.6%in patients vs. 27.8% in normal cases), And the ulnar loop didn't show any significant difference [43].

In summary, it can be concluded that arches and loop patterns may be considered to be a factor in identifying the predisposition to leukemia development in women in Iraq.

## 7 Pituitary tumors

Pituitary tumors are lesions of the central nervous system [44]. In 2016 Gradiser M. et al. Studied environmental and hereditary factors which influence pituitary tumors in 126 cancer patients of both genders with non-functional and functional (60 and 66, respectively) pituitary tumors and the 400 control individuals who clinically were healthy at Mercy University Hospital in Ireland. As a result, in functional tumors in males, the ulnar loop increased by 69.0% in patients versus 56.2% in normal individuals, and arch patterns increased (8.5%) compared with normal individuals (5.3%). However, whorl patterns decreased (20.0% patients vs. 33.9% normal) in functional tumors in females. Ulnar loop increased (60.9%) compared to normal (59.9%). Besides, arch patterns showed a higher percentage (9.1%) in comparison with normal females (4.6%), and whorl patterns in patients showed decreases (26.7%) in comparison with normal females (31.9%) [45].

In this study, we mainly focused on three main patterns of the whorl, arch, and loop in ≥6 digits of hands in 6 different cancer groups with the most research available in the literature. The overall results are tabulated in Table 1.

**Table 1:** Comparison of fingerprints in different cancer.

Cancer Type	Percentage of each fingerprint			Total patients and control cases	Predominant pattern	Population	Results	Year	Ref
	Loop*	Arch	Whorl						
**Breast cancer**	**-**	**-**	P:48.7% N:27.5%	P:154 C:308	**-**	Iran	the presence of 6 or more Whorls is associated with statistically significant breast cancer	2006	[16]
	P:66.5% N:59.2%	P: 3.2% N: 6.8%	P:30.3% N:34.0%	P:1000 C:1000	Loop	India	The total loops and left-hand loops are statistically significant predictors of breast cancer	2010	[19]
	**-**	**-**	P:53.3% N:23.33%	P:30 C:30	**-**	India	The presence of≥6 whorls and the total number of whorls is a statistically significant predictor of breast cancer	2012	[20]
	P:36.8% N:50.4%	P:39.8% N: 16.2%	P:20.2% N: 30.8%	P:500 C:500	Radial loop	India	The Whorl, Arch, and Radial loop in both hands and Ulnar loop pattern count in right hands figures are significantly associated with breast cancer	2013	[21]
	P:63% N:78.5%	P:29% N:25.2%	P:23% N:14.4%	P:100 C:132	**-**	Bosnia and Herzegovina	The presence of≥6 whorls and loops is not significantly associated with breast cancer	2018	[22]
	P:50.40% N:65.00%	P:3.80% N:6.40%	P:42.80% N:23.80%	P:82 C:60	**-**	Indonesia	breast cancer patients tend to have a whorl fingerprint pattern	2021	[25]
**Cervical cancer**	P: 52.8% N:7.3%	–	–	P:72 C:72	Ulnar loops	India	The total Ulnar loops, Radial loops, and Whorls has significantly associated with cervical cancer	2018	[29]
**Oral cancer (OSSC)**	P: 50% N:12%	P: 30% N: 20%	P:20% N:68%	P:10 C:25	Whorl	India	Arches and loops were more frequent in oral cancer cases	2020	[32]
	P:58.6% N:49.6%	P:70% N: 2.0%,	P:34.4% N:48.4%	P:60 C:60	Ulnar loops	India	Whorl, Arches, and Ulnar loops count are significantly associated with Oral squamous cell carcinoma	2013	[33]
	P:46.4%, N:48.15%	P:6.7% N:5.0%	P:46.8% N:46.9%	P:70 C:70	Ulnar Loop	Iran	The arch pattern was significantly higher in the cancer group	2022	[34]
	P: 60% N: 30%	P:7% N:2%	P:32% N:68%	P:30 C:30	Loop	India	Arch and loop patterns were more frequent in the case group. Whorl patterns were more frequent in the control group	2022	[35]
	P:33.3% N:30.6%	P:60.7% N:28.6%	P:29.0% N:40.5%	P:30 C:30	Loop	India	The arch pattern was more frequent in the case group. Whorl patterns were more frequent in the control group	2022	[36]
**Prostate cancer**	P:1.32% N:1.07%	P:17.11% N:13%	P:37.17% N:30.67%,	P:30 C:30	Ulnar loop	India	Whorl, Arch, and radial loop patterns were more frequent in the cases group	2020	[38]
**Gastric cancers**	P:27.5% N:46.2%	P: N:	P:54.2% N:44.5%	P:153 C:299	**-**	Iran	Whorl and loop patterns are significantly associated with Gastrointestinal cancers	2017	[40]
**Leukemia**	P:15% N:29%	P:7% N:4%	P:26% N:15%	P:24 C:24	Loops	India	Radial, double, and central pocket loops and tented arches patterns are higher in the control group	2011	[42]
	In male					Iraq	Arch and Ulnar loops were more frequent in male patients. Whorls are more frequent in the male control group. Arch and Whorl patterns were more frequent in female patients with leukemia. Ulnar and radial loops are more frequent in the control group.	2017	[43]
	P: 42.2% N: 28.8%	P: 12.2% N: 6%	P: 32.4% N: 38.8%	P:50 C:50	Whorl				
	In female:								
	–	P: 14.4% N: 8%	P: 36.2% N: 28.8%	P:50 C:50	Ulnar loop				
**Pituitary tumors**	In males with functional tumor					Ireland	whorl, ulnar loop, radial loop, and arch patterns of the right hand and sum of both hands were significantly different comparing functional tumor groups and controls in both male and female groups	2016	[45]
	P: 69.0% (from 20) N: 56.2% (from 30)	P: 8.5% (from 20) N: 5.3% (from 30)	P: 20.0% (from 20) N: 33.9% (from 30)	P:20 C:200	Ulnar Loop				
	In females with functional tumor								
	P(ulnar): 60.9% (from 30) N(ulnar): 59.9% (from 46)	P: 9.1% (from 30) N: 4.6% (from 46)	P: 26.7% (from 30) N: 31.9% (from 46)	P:46 C:200	Ulnar Loop				

P = patients N= Normal individuals * = Ulnar loop.

Most of the studies that investigated dermatoglyphics on cancers have been conducted in India. There are few studies conducted in western countries in this regard. It could be useful to conduct more research in other countries, specifically in western countries.

## 8 Conclusion

The dermatoglyphic analysis could be a helpful tool for early genetic diagnosis. It could be a valuable tool for identifying people with a particular genetic predisposition to develop certain genetic disorders. Some patterns could alert us about fatal diseases that require more attention. These alerts could lead to a decline in the death rate with an early cancer diagnosis and taking proper care. However, few studies have been done on dermatoglyphics and cancers, so it requires further comprehensive and multi-centric studies in different populations and ethnicities to conclude accurately.

## Author contribution statement

All authors listed have significantly contributed to the development and the writing of this article.

## Funding statement

This research did not receive any specific grant from funding agencies in the public, commercial, or not-for-profit sectors.

## Data availability statement

No data was used for the research described in the article.

## Declaration of competing interest

The authors declare that they have no known competing financial interests or personal relationships that could have appeared to influence the work reported in this paper.
